# Carbon, Glass and Basalt Fiber Reinforced Polybenzoxazine: The Effects of Fiber Reinforcement on Mechanical, Fire, Smoke and Toxicity Properties

**DOI:** 10.3390/polym12102379

**Published:** 2020-10-15

**Authors:** Nick Wolter, Vinicius Carrillo Beber, Anna Sandinge, Per Blomqvist, Frederik Goethals, Marc Van Hove, Elena Jubete, Bernd Mayer, Katharina Koschek

**Affiliations:** 1 Faculty of Production Engineering, University of Bremen, Badgasteiner Straße 1, 28359 Bremen, Germany; nick.wolter@ifam.fraunhofer.de (N.W.); bernd.mayer@ifam.fraunhofer.de (B.M.); 2 Fraunhofer Institute for Manufacturing Technology and Advanced Materials IFAM, Wiener Strasse 12, 28359 Bremen, Germany; vinicius.carrillo.beber@ifam.fraunhofer.de; 3 RISE Research Institutes of Sweden, Brinellgatan 4, SE-50462 Boras, Sweden; anna.sandinge@ri.se (A.S.); per.blomqvist@ri.se (P.B.); 4 DTU, Technical University of Denmark, 2800 Kongens Lyngby, Denmark; 5 CENTEXBEL, Technologiepark 70, 9052 Gent-Zwijnaarde, Belgium; frg@centexbel.be (F.G.); mvh@centexbel.be (M.V.H.); 6 CIDETEC, Basque Research and Technology Alliance (BRTA), P° Miramon 196, 20014 San Sebastian, Spain; ejubete@cidetec.es

**Keywords:** polymer-matrix composites, fibers, mechanical properties, thermal properties, polybenzoxazine, fire, smoke and toxicity

## Abstract

Bisphenol F and aniline-based benzoxazine monomers were selected to fabricate basalt, glass and carbon fiber reinforced polybenzoxazine via vacuum infusion, respectively. The impacts of the type of fiber reinforcement on the resulting material properties of the fiber reinforced polymers (FRPs) were studied. FRPs exhibited a homogenous morphology with completely impregnated fibers and near-zero porosity. Carbon fiber reinforced polybenzoxazine showed the highest specific mechanical properties because of its low density and high modulus and strength. However, regarding the flammability, fire, smoke and toxicity properties, glass and basalt reinforced polybenzoxazine outperformed carbon fiber reinforced polybenzoxazine. This work offers a deeper understanding of how different types of fiber reinforcement affect polybenzoxazine-based FRPs and provides access to FRPs with inherently good fire, smoke and toxicity performance without the need for further flame retardant additives.

## 1. Introduction

Fiber-reinforced polymers (FRP) exhibit unique material properties, specifically high stiffness-to-weight ratios and excellent corrosion resistance, compared to other materials, e.g., metals [1,2,3]. In some cases, combustible FRPs can replace non-combustible materials such as steel. Hence, the fire, smoke and toxicity (FST) performance of composites becomes a top priority to maintain high fire safety levels. However, most commonly used FRPs are flammable materials that easily ignite and burn when exposed to fire, emitting highly dense and toxic smoke and gases [4,5,6]. One way to improve FRP’s FST properties is to avoid a high content of combustible polymers by introducing non-combustible fiber reinforcement made from, e.g., glass, Kevlar, poly(p-phenylene-2,6-benzobisoxazole), carbon or basalt [7,8,9,10].

Among these three types of fibers, carbon fibers exhibit the lowest density coupled with the highest tensile strength and modulus, but they exhibit the lowest maximal application temperature, highest costs and comparably low elongation at break [11]. Furthermore, carbon fibers directly exposed to fire tend to oxidize in the presence of oxygen at temperatures exceeding 350–450 °C. The rate of oxidation depends on the precursor (pitch or polyacrylonitrile) used for carbon fiber production, the presence of impurities or submicron-sized voids in the graphitic structure and the fire conditions (temperature, oxygen content, gas velocity) [6,12]. However, oxidation usually does not occur extensively in composites because of the formation of a char layer upon polymer decomposition that protects the carbon fibers and prevents oxygen transfer to them [6,13].

Mineral fibers from glass or basalt exhibit higher service temperatures of roughly 800 °C, melting points ranging from 1050 to 1700 °C, and excellent fire barrier properties up to 1200 °C [14,15]. Basalt fibers show the highest thermal and fire properties compared to carbon and glass, meaning they are often used in thermal insulation and passive fire protection applications. Furthermore, basalt fibers are highly chemically stable, resistant against weather, alkaline and acid exposure, and exhibit similar or better thermal and mechanical properties than glass fibers [16,17,18].

In view of fire safety, FRPs’ FST properties can also be improved through the implementation of thermally stable polymers as the matrix component. Polybenzoxazines show good inherent flame resistance, high heat resistance, high glass transition temperatures, excellent mechanical performance and chemical stability when compared to other thermosets such as epoxies [19,20] or phenolics [21]. They have been studied as matrices in FRPs with different types of fiber reinforcement, such as carbon [22,23,24,25,26], glass [27,28,29,30,31], natural [32,33,34] and quartz [35,36] fibers. However, within these studies, different types of benzoxazine monomers and manufacturing techniques were used for the FRP manufacture [37]. Therefore, little is known about the effect of the type of fiber reinforcement on the mechanical performance correlated with the fire, smoke and toxicity properties of fiber-reinforced polybenzoxazines.

The present work aims to provide carbon, glass and basalt fibers as reinforcements for bisphenol F and aniline-based polybenzoxazine (PBF-a). A deeper understanding of how fiber reinforcement affects the physical, mechanical and FST properties of PBF-a based FRPs will offer access to thermally stable FRPs with inherent good FST performance without the need for further flame retardant additives.

## 2. Materials and Methods

### 2.1. Materials

Bisphenol F and aniline-based benzoxazine (BF-a) (Araldite MT 35700) from Huntsman Advanced Materials GmbH (Basel, Switzerland) was used. Carbon (twill 2/2, 200 g/m²) and glass (twill 2/2, 390 g/m²) were obtained from MC Technics (Visé, Belgium). Basalt fabric (atlas, 350 g/m²) was supplied by Basaltex (Wevelgem, Belgium). For the glass and basalt fibers, the sizing was organosilane-based, whereas the carbon fibers exhibited an epoxy-based sizing. The carbon fibers exhibited a smaller diameter of 7 µm and a lower density of 1.76 g/cm^3^, compared to glass (9 µm and 2.54 g/cm^3^) and basalt (13 µm and 2.67 g/cm^3^) fibers.

### 2.2. Methods

#### 2.2.1. 1-Step Vacuum Infusion FRP Manufacturing

Carbon, basalt and glass fiber reinforced PBF-a were manufactured using the vacuum infusion technique, in which fiber impregnation and curing were conducted in a single step. This FRP manufacturing method is referred to here as 1-step vacuum infusion and was performed as follows:

BF-a monomers were heated to 140 °C in a convection oven (Heraeus Kelvitron T, Hanau, Germany) and degassed in an automated degassing system (Thinky mixer ARV-930, Laguna Hills, CA, USA) (parameters: rotational velocity of 14000 rpm; vacuum of 0.5 kPa; degassing time of 4 min). Depending on the fiber type, varying numbers of fabric plies with the dimensions 600 × 850 mm^2^ (basalt and glass) and 300 × 300 mm^2^ (carbon) were laid in a 0° configuration on an aluminum plate and sealed in a double vacuum bag setup. The 1-step vacuum infusion was performed in a convection oven (Vötsch VTL 100/150 from Vötsch Industrietechnik GmbH, Reiskirchen, Germany) at a temperature of 140 °C. After complete wetting, the temperature was immediately increased in order to start the curing process that consisted of the following steps: heating to 170 °C over 15 min, maintaining at 170 °C for 3 h, increasing the temperature to 180 °C over 15 min, maintaining at 180 °C for 2 h, increasing the temperature to 200 °C over 15 min, and maintaining at 200 °C for 4 h before cooling to room temperature. The fully cured specimens were removed from the oven at room temperature.

#### 2.2.2. Material Characterization

Quasi-static tensile testing was performed according to DIN EN ISO 527-4 using specimen type 3 with the dimensions 4 × 250 × 25 mm^3^ and a testing speed of 2 mm/min with a RetroLine 100 kN from a Zwick/Roell testing machine. Interlaminar shear strength testing was conducted on the aforementioned machine with a testing speed of 1 mm/min following DIN EN ISO 14130. A sample size of 4 × 40 × 20 mm^3^ was selected and due to different thicknesses, the selected span length differed between the FRPs (21.5 mm for CFRP, 18 mm for BFRP, 21 mm for GFRP). Flexural testing of the composites was performed according to DIN EN ISO 14125. Type 3 specimens were selected for BFRP and GFRP (dimensions 4 × 120 × 15 mm^3^), and the testing was performed on a Zwick 100 kN with a testing speed of 2.5 mm/min and a distance between supports of 80 mm. For CFRP, type 4 specimens were used (dimensions of 4 × 200 × 15 mm^3^) with a testing speed of 10 mm/min and a distance between the supports of 160 mm. Flexural and ILSS testing were performed using rolls for supports with a diameter of 10 mm. Quasi-static mechanical testing was performed in five-fold repetition.

Fatigue testing was performed in a Schenk Typ PC 400 M with a load of ±400 kN following ISO 13003 guidelines. The specimen dimensions were 185 × 25 × 4 mm^3^ with 80 mm bonded tabs that yielded an unsupported length of 25 mm between the grips of the fatigue loading machine. The cyclic loading during the fatigue testing had a uniform stress with R-values of −1.0 (compression-tension), 0.1 (tension-tension) and 0.5 (tension-tension) applied with a frequency of 10 Hz.

Optical microscopy images were taken with a Keyence VHX-1000 microscope from Keyence (Neu-Isenburg, Germany), while a JSM-7600F FEG-Scanning Electron Microscope from JEOL (Akishima, Japan) with a resolution of 2 nm at 2 and 10 kV was used for scanning electron microscopy (SEM) imaging under a high vacuum. Pre-coating of the FRPs with a 4 nm thick platinum/palladium layer via sputtering was necessary to avoid electrical charging during SEM. The densities of the specimens were measured according to EN ISO 1183-1, and fiber volume content (FVC) measurements were performed following ASTM D3171-15 in three-fold repetitions. Thermogravimetric analysis (TGA) measurements were carried out under ambient and inert (nitrogen) atmospheres using a TGA Q5000 calorimeter from TA Instruments (New Castle, DE, USA). Specimen weights of 40–60 mg, a heating rate of 10 °C/min and a temperature range of 20 °C to 800 °C/1000 °C were used for testing.

A limiting oxygen index (LOI) test was conducted according to the EN ISO 4589-2 (specimen type IV, dimensions 100 × 10 × 4 mm^3^) using an LOI by Fire Testing Technology. Cone calorimeter testing according to ISO 5660-1 was performed with a cone calorimeter from Fire Testing Technology, using horizontally mounted specimens with a heat flux of 50 kW/m^2^ and a test duration of 20 min. In the spread of flame test, the specimen was mounted vertically and exposed to a pilot burner and radiation from a radiation panel according to ISO 5658-2, using lateral flame spread equipment from Fire Testing Technology. The smoke chamber testing of FRPs was conducted in accordance with EN ISO 5659-2 with a smoke chamber from Fire Testing Technology coupled with additional FTIR equipment (Thermo Scientific Antaris IGS Analyzer, Waltham, MA, USA) from Nicolet for toxic gas analysis according to EN 45545-2, Annex C. Tests were performed with an irradiance level of 50 kW/m^2^ without a pilot flame and the test duration was 10 min. Sampling of the smoke gases was performed with FTIR at 4 and 8 min for toxicity measurements. Triplicate tests were performed for GFRP and BFRP, while only duplicate tests were performed for CFRP and neat PBF-a for the aforementioned tests.

## 3. Results and Discussion

**Figure 1:**
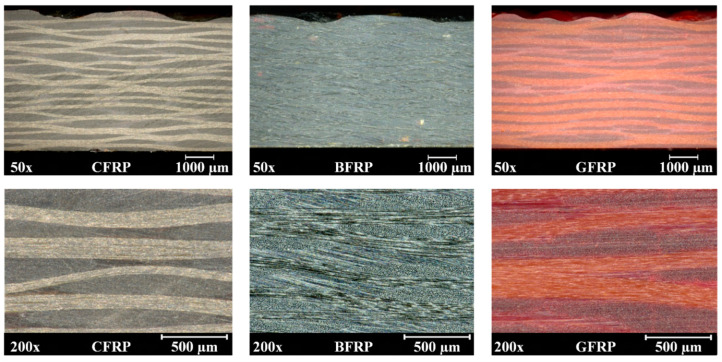
Microscopic images of cross-sections of carbon, glass and basalt fiber reinforced PBF-a with magnifications of 50× and 200×.

A total of 20 plies of carbon fiber (CFRP), 15 plies of basalt fiber (BFRP) and 14 plies of glass fiber (GFRP) fabrics were used in a 0° configuration for the 1-step vacuum infusion with BF-a, respectively, in order to obtain composites with similar thicknesses. All FRPs exhibited thicknesses in a similar range of about 3.9–4.2 mm. The CFRP exhibited a fiber volume content (FVC) of around 60 vol%, whereas the BFRP and GFRP exhibited slightly lower ones of roughly 57 vol% and 54 vol%, respectively. The BFRP and GFRP composites exhibited the highest density of 1.9 g/cm^3^, while the CFRP had the lowest density of 1.5 g/cm^3^ (Table A1). Microscopic images of cross-sections displayed a homogeneous morphology with well-penetrated fibers and near-zero porosity for all FRPs (Figure 1).

### 3.1. Mechanical Properties

#### 3.1.1. Quasi-Static Mechanical Properties

**Figure 2:**
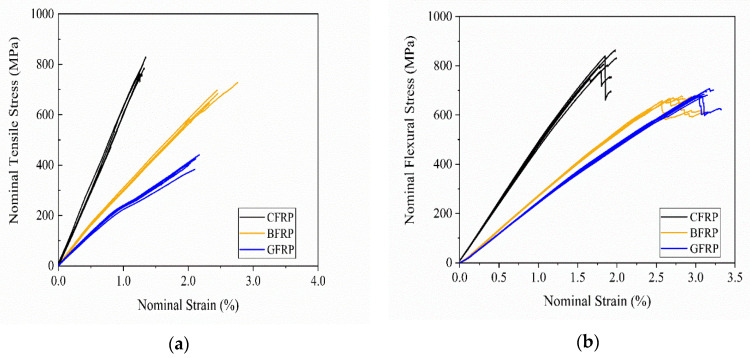
Stress-strain curves of quasi-static tensile (**a**) and flexural (**b**) testing.

The impacts of the type of fiber reinforcement on the quasi-static tensile, flexural and interlaminar mechanical properties were determined by measuring the fiber-dominated and fiber-matrix-interface-dominated properties. Figure 2 depicts the nominal stress-strain curves of the quasi-static tensile (a) and flexural (b) testing.

**Figure A1:**
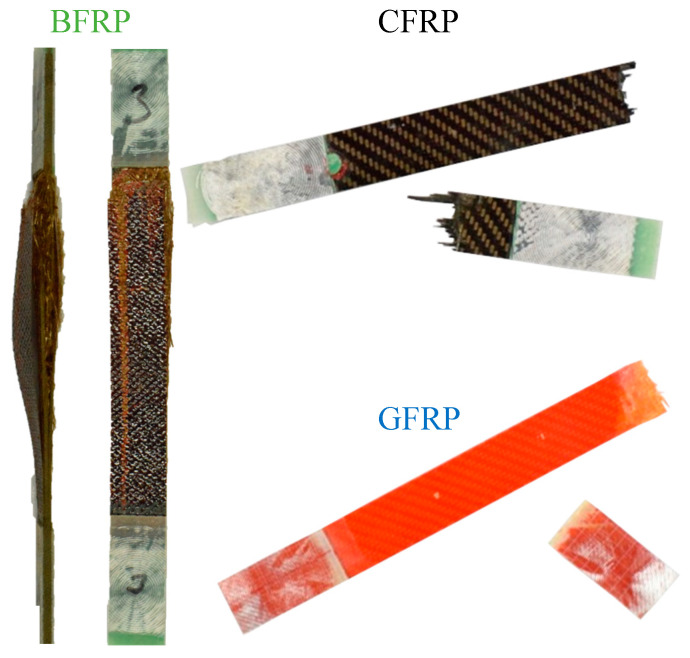
Fracture patterns of carbon, glass and basalt fiber reinforced PBF-a after tensile testing.

Nearly linear nominal stress-strain relationships that persisted until failure were obtained for all FRPs in tensile and flexural testing with one exception; namely, the GFRP composites showed a linear stress-strain relationship until there was 1% nominal strain, followed by a second linear stress-strain increase until failure occurred in tensile testing. This may be explained by a slippage of the tabs that were adhesively bonded to the surface of the GFRP specimens or by a failure of single layers of the composite due to, e.g., deviating fiber orientations of single layers. Furthermore, the GFRP failed in the tabbed area clamped by the fixture during testing (Figure A1). Inhomogeneous stress distribution and stress concentrations can cause a failure during tensile testing in this region, which may have led to lower tensile strength and strain at failure in comparison to failure events in the center of the composite, where a homogenous stress distribution dominated.

**Figure 3:**
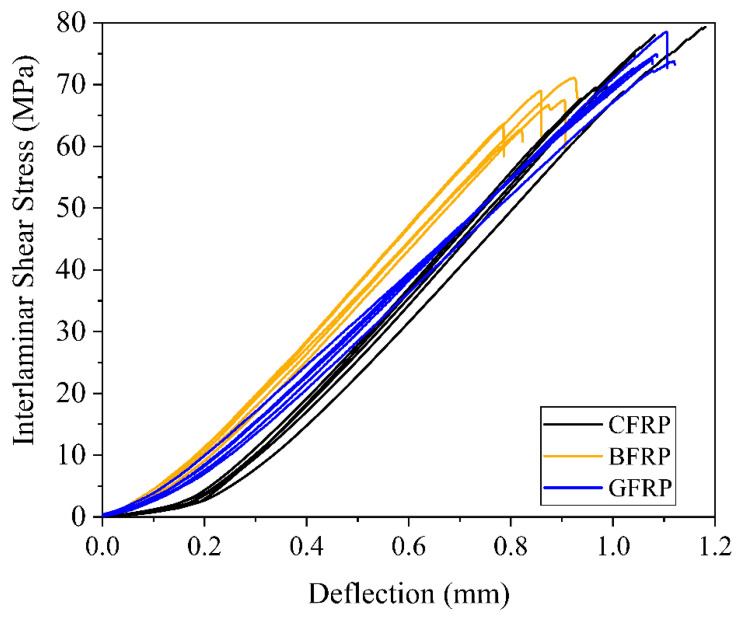
Interlaminar shear stress—deflection curves of apparent interlaminar shear strength testing.

The CFRP exhibited the highest tensile and flexural modulus and strength, whereas the BFRP and GFRP exhibited the highest strain at failure (Table A2). This is in accordance with the mechanical properties of the neat fiber reinforcement as carbon fibers are known for their high strength and stiffness, whereas fibers from glass or basalt exhibit high elongation at break. FRPs’ tensile and flexural mechanical properties are fiber-dominated properties, whereas the apparent interlaminar shear strength (ILSS) display fiber-matrix-interface-dominated properties. Figure 3 presents the interlaminar shear stress—deflection curves.

All FRPs exhibited high ILSS values, meaning good compatibility between the fiber, fiber sizing and matrix. GFRP and CFRP showed comparable ILSS values in the range of 75 MPa, whereas the one for BFRP remained lower than the aforementioned, which may be explained by the basalt fiber’s larger diameter of 13 µm in comparison to the 9 µm and 7 µm for glass and carbon fibers, respectively. In view of that, larger fiber diameters provide smaller surface contact between the fiber and the matrix, which leads to lower interlaminar shear strength. Previous studies have shown a similar relationship for, e.g., glass fiber reinforced polyesters [38].

Glass and basalt fibers contain an organosilane while carbon fibers exhibit an epoxy-based sizing. The results indicate that the different sizings did not affect the fiber matrix interaction. PBF-a showed good compatibility with different types of sizings.

#### 3.1.2. Fatigue Mechanical Properties

Under operation conditions, fiber-reinforced structures are exposed to cyclic loads (e.g., vibration), making fatigue strength a highly relevant property in the selection of materials. Thus, the polybenzoxazine-based FRPs were tested under cyclic loading to assess the influence of the type of fiber reinforcement on their fatigue strength.

**Figure 4:**
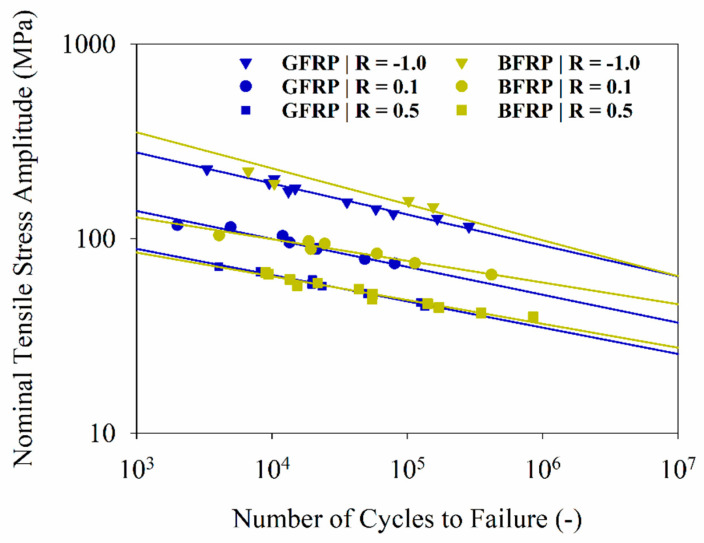
Fatigue testing results of basalt fiber (BFRP) and glass fiber (GFRP), SN curves for R = −1.0, 0.1 and 0.5.

Due to the large number of samples and lengthy testing time, the fatigue characterization was limited to BFRP and GFRP with similar FVCs and numbers of plies. Stress amplitudes were determined to capture lifetimes (*N_f_*) from the low to high cycle range (10^3^ < *N_f_* < 10^6^). The experimental fatigue data points were fitted using Basquin’s law [39]. The S–N curves are shown in a double-log chart with a 50% probability of failure (Figure 4).

The parameters of the S–N curves indicated a good correlation of linear regressions (*rsq* > 0.94). The deleterious effect of the mean stress increase can be observed for BFRP and GFRP, as the fatigue strength is reduced with an increasing stress ratio, with the highest strength obtained for R = −1 and the lowest for R = 0.5 (Table A3). For the same stress amplitude, the higher the value of R, the higher the effect of mean stress. BFRP had a fatigue strength similar to or higher than GFRP for all values of R, which is consistent with their respective tensile quasi-static results; for example, BFRP’s fatigue strength at 10^6^ cycles was 59 MPa, whereas that for GFRP was 51 MPa. The fatigue strengths of polybenzoxazine-based FRPs were in the same range as basalt (53 MPa) or glass (54–91 MPa) fiber reinforced epoxies from the literature for similar stress ratios and numbers of cycles [40,41].

### 3.2. Thermal and Thermal-Oxidative Decomposition Properties

**Figure 5:**
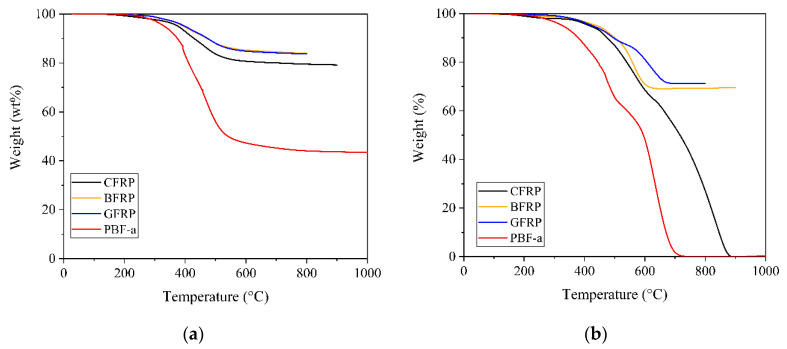
Weight-temperature curves of neat PBF-a as well as carbon, glass and basalt fiber reinforced PBF-a under (**a**) nitrogen and (**b**) ambient atmospheres.

Thermogravimetric analysis was conducted on the neat polymer and on the FRPs using inert and ambient atmospheres to study the influence of the type of fiber reinforcement on the thermal and thermal-oxidative decomposition, respectively. The weight-temperature curves of PBF-a and FRPs under (a) nitrogen and (b) ambient atmospheres are depicted in Figure 5, and the decomposition temperatures at 2 and 10% mass loss (T_2%_ and T_10%_) and the char yield at 800 °C are summarized in Table A4.

The neat PBF-a and FRPs showed a thermally induced and temperature-dependent decomposition in both atmospheres. Upon addition of fiber reinforcement, the decomposition temperatures at 2 and 10% weight loss and char yields increased. Basically, the presence of fiber reinforcement decreased the amount of polymeric material that can volatilize during TGA testing.

Additionally, GFRP and BFRP exhibited higher char yields than CFRP in both atmospheres. For instance, GFRP and BFRP retained up to 70–71 wt%, whereas CFRP only yielded 27 wt% at 800 °C under ambient atmosphere. Mineral fiber reinforced PBF-a was more stable against thermal and thermal-oxidative decomposition than PBF-a reinforced with carbon fibers.

Furthermore, CFRP subsequently decomposed completely with increasing temperatures (900 °C) under ambient atmosphere. PBF-a reinforced with basalt or glass fibers did not show further weight losses between 800 and 1000 °C. The effect of carbon fibers on the char yield under thermal-oxidative decomposition can be associated with the oxidation of carbon fibers in the presence of oxygen. It was previously reported that carbon fibers decompose under high temperature exposure in the presence of oxygen, resulting in low or even absent char yields during TGA [42]. In contrast, basalt and glass fibers were shown to exhibit low weight losses at temperatures up to 1000 °C [43].

### 3.3. Reaction-to-to Fire Properties

#### 3.3.1. Flammability, Heat Release Properties and Spread of Flame

Limiting oxygen index testing was performed to assess the flammability behavior of neat PBF-a and FRPs, taking oxygen index (OI) values into account. Neat PBF-a showed an OI of 26.8%, which was increased to 44–54% upon fiber reinforcement. The FRPs outperformed neat PBF-a in terms of flammability behavior, but the type of fiber reinforcement itself proved to have an influence on the OI values. BFRP showed the highest OI of 54.3 ± 0.2%, followed by GFRP with 53.7 ± 0.2% and CFRP with 44.0 ± 0.3%. The introduction of mineral fibers into PBF-a led to higher OIs than carbon fibers and, thus, BFRP and GFRP were less flammable than CFRP. The PBF-a based FRPs exhibited high OI values and thus low flammability in comparison to other glass fiber reinforced thermosets, such as epoxy (OI of 38%), phenolic (OI of 54%) and bismaleimide (OI of 60%) [44].

**Figure 6:**
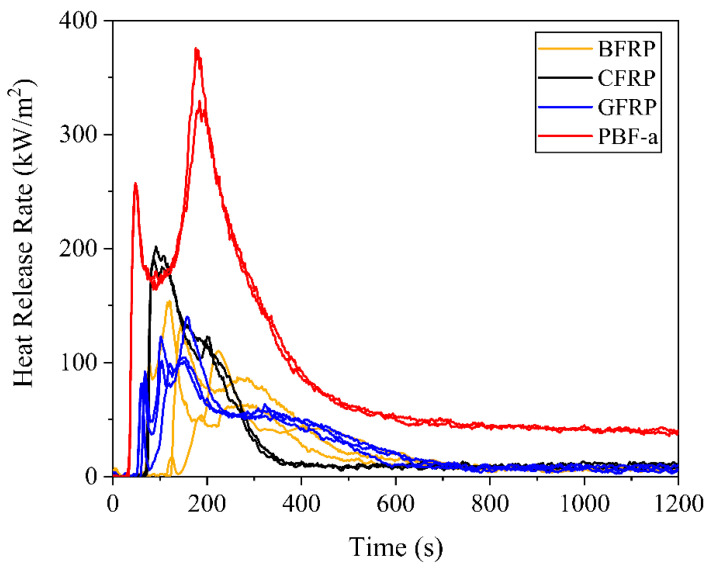
Heat release rate as a function of time for the three types of composites and non-reinforced PBF-a.

In addition to flammability testing, reaction-to-fire testing was performed following EN 45545-2, which includes requirements for heat release, spread of flame and production of smoke as well as toxic gases for railway application. The heat release properties of the neat PBF-a and FRPs were assessed by cone calorimeter tests (Figure 6). Time to ignition (TTI), peak heat release rate (PHRR) and total heat release (THR) were measured during the tests and the Maximum Average Rate of Heat Emission (MARHE) were calculated based on measured heat release (Table A6).

All FRPs showed a typical reaction-to-fire behavior of charring materials [45] as the heat release rate (HRR) time curves displayed one peak after roughly 50–250 s, followed by a maximum peak (PHRR) and a decrease in the HRR until the end of the test. The peak can be attributed to the ignition of combustible pyrolysis gases that were released due to the thermal decomposition of PBF-a. Throughout the test, the amount of non-combustible residues increased, which led to a decrease of both gas transfer from the pyrolysis zone to the surface and heat transfer from the surface to the pyrolysis zone. Thus, the HRR converged to a comparably low point below 15 kW/m^2^ after roughly 400 s for CFRP and 700 s for BFRP and GFRP. Neat PBF-a showed two peaks in the HRR time curves, an earlier ignition (TTI) and higher PHRRs, as well as a convergency to a comparably high HRR of 50 kW/m^2^ after 600 s.

The addition of fiber reinforcement to neat PBF provided substantial improvements with respect to TTI as well as the heat release properties. This is largely due to the reduced PBF-a content caused by the presence of fibers. The different types of fiber reinforcement yielded comparably high char yields in the combustion zone and, therefore, provided an effective heat and gas barrier.

Furthermore, the type of fiber reinforcement had a significant influence on the above-mentioned properties. GFRP exhibited the shortest TTI with 59 s, followed by CFRP (32% longer) and BFRP (41% longer). The CFRP exhibited the highest PHRR and MARHE of 196 kW/m^2^ and 95 kW/m^2^, respectively, whereas the total heat release (THR) was similar for all FRPs. Thus, mineral fibers from basalt or glass tend to flatten the heat release rate time curves, yielding lower PHRR and MARHE values, while the THR remains similar. This implies that for the CFRP, the same heat was released in less time, which might lead to a faster development of a fire.

The PHRR and THR values from the literature, based on cone calorimeter measurements according to ASTM E1354 with a heat flux of 50 kW/m^2^, demonstrate that all PBF-a based FRPs exhibited low heat release:

- PHRR: phenolic (73 kW/m2) < GFRP (121 kW/m2) < CFRP (196 kW/m2) < epoxy (350 kW/m2) [44]
- THR: phenolic (18 MJ/m2) < BFRP (29 MJ/m2) < CFRP (35 MJ/m2) < epoxy (48 MJ/m2) [44].

In addition to cone calorimetry, spread of flame testing was conducted with the most promising materials in terms of the heat release properties (BFRP and GFRP). The Critical Flux at Extinguishment (CFE) and average heat for sustained burning are summarized in Table 1. For both parameters, the higher the value, the less prone the material is to flame spreading. BFRP and GFRP showed CFEs in the same range, but the standard deviation of GFRP is quite high. GFRP exhibits a higher value for average heat for sustained burning compared to BFRP.

BFRP and GFRP showed CFE values in the spread of flame testing in the same range as glass fiber reinforced epoxies for railway applications from the literature and, thus, can be characterized to have a low flame spread:

CFE: glass fiber reinforced epoxy (30–38 kW/m^2^) ≤ GFRP (34 kW/m^2^) ≤ BFRP (39 kW/m^2^) [46].

#### 3.3.2. Smoke and Toxicity Properties

**Figure 7:**
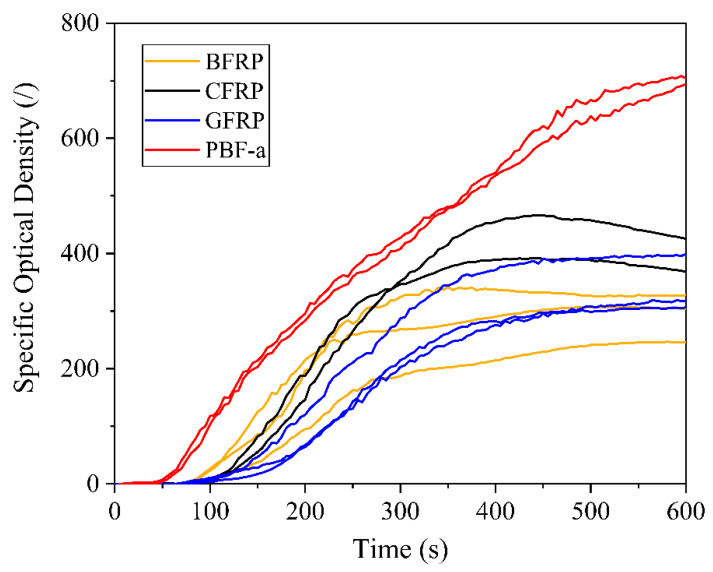
Specific optical density (Ds) as a function of time.

**Figure A2:**
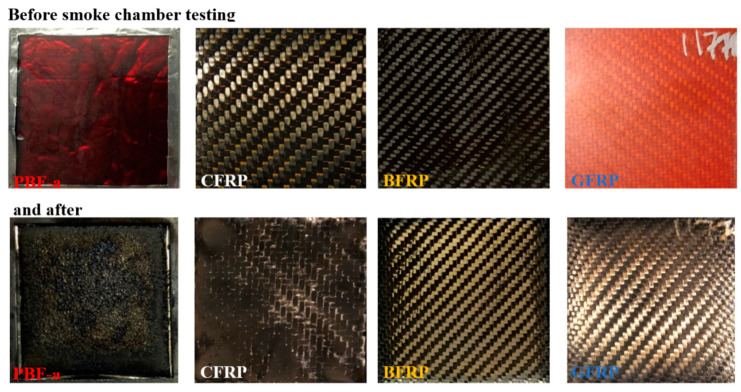
Neat PBF-a as well as carbon, glass and basalt fiber reinforced PBF-a before and after smoke chamber testing.

The effect of the type of fiber reinforcement on the smoke density and toxicity of PBF-a was determined by performing smoke chamber testing coupled with FTIR equipment. Figure 7 depicts the specific optical density (D_s_) as a function of time for the three FRP configurations and the neat PBF-a, while Figure A2 presents pictures of the specimens before and after smoke chamber testing. Furthermore, smoke density properties, namely the maximum value of specific optical density (D_s,max_), specific optical density at 4 min (D_s_(4)) and the cumulative value of specific optical density of smoke in the first 4 min (VOF_4_), are shown in Table A7. The smoke gases accumulate upon test duration, decreasing light transmission and therefore increasing D_s_.

The D_s_ for FRPs displayed an almost sigmoid curve progress that started after approximately 100 s, increased upon testing time, and reached a plateau after roughly 400–500 s. A deviation from the stabilization towards a constant value was obtained for CFRP as the D_s_ showed evidence of a declining trend after reaching D_s,max_.

The addition of carbon, basalt and glass fiber reinforcement to PBF-a provided a substantial decrease in the smoke density properties. The improvements can be attributed to the reduced PBF-a content and the presence of non-combustible fibers. In view of that, the different types of fibers themselves impacted the smoke density properties. CFRP emitted a greater amount of smoke gases with a higher optical density than BFRP (decreased by 16%) or GFRP (decreased by 45%) at 240 s (Ds(4)). Additionally, CFRP exhibited a higher D_s,max_ value compared to BFRP and GFRP. VOF_4_ represents the specific optical smoke density over the first 4 min of the test. In view of that, GFRP exhibited lower smoke emissions when compared to BFRP and CFRP. Mineral fiber reinforced PBF-a exhibited lower smoke emissions compared to CFRP, substantially improving the smoke density properties of PBF-a.

The conventional index of toxicity (CIT_G_) was measured using FTIR equipment and the detected gas species are listed in Table A8. CFRP and BFRP exhibited CIT_G_ values at 8 min in a similar range, namely 0.26 ± 0.01 and 0.20 ± 0.13, respectively, whereas neat PBF-a and GFRP showed lower CIT_G_ values of 0.14 ± 0.01 and 0.06 ± 0.01, respectively. These differences can be explained by the different gas species during FTIR gas analysis. The detected gases for all materials included carbon dioxide (CO_2_), carbon monoxide (CO) and hydrogen cyanide (HCN). Nitrogen oxides (NO_x_) were only detected for CFRP and BFRP. The highest CO_2_ content was obtained for CFRP, followed by BFRP (two-fold decrease) and neat PBF-a (21-fold decrease) as well as GFRP (32-fold decrease). This can be explained by varying flaming combustion conditions between the different tests after 8 min as CO_2_ is only produced during well-ventilated combustion after ignition [47]. CFRP ignited in all tests, while BFRP showed non-flaming combustion in one of the tests and no ignition occurred for GFRP or neat PBF-a (ignition after 550 s and thus after FTIR measurements). Furthermore, carbon fibers tend to thermo-oxidatively decompose in the presence of oxygen and high temperatures, which contributes to the release of CO and CO_2_. Neat PBF-a showed the highest CO-content of all tested materials in the FTIR gas analysis after 8 min, which can be attributed to both its late ignition and the accumulation of non-burned pyrolysis gases.

Despite its positive effect on specific optical density, the presence of fiber reinforcement did not always provide improvement in view of the smoke toxicity of PBF-a. CIT_G_ only decreased in the case of GFRP, whereas BFRP and CFRP showed higher values than neat PBF-a.

However, the PBF-a based FRP´s specific optical density and toxicity of smoke gases are lower than those of glass fiber reinforced epoxies for railway applications presented in the literature:

- VOF4: glass fiber reinforced epoxy (800–1011) > PBF-a (589) > CFRP (287) > BFRP (280) > GFRP (150) [46]
- CITG after 4 min: glass fiber reinforced epoxy (0.3–0.33) > CFRP (0.08) > BFRP (0.07) > PBF-a (0.02) > GFRP (0.01) [46].

## 4. Conclusions

This work provides a comparative investigation of the effects of the type of fiber reinforcement on FRP´s physical, mechanical, flammability and FST properties. Basalt, glass and carbon fiber reinforced PBF-a were fabricated via 1-step vacuum infusion, producing well-penetrated and near-zero porosity FRPs. These results show that carbon fibers yielded the lowest density and highest mechanical properties, while mineral fibers exhibited better thermal, flammability and FST properties. Furthermore, it was shown that PBF-a based composites exhibit good inherent FST properties that are better than or similar to glass fiber reinforced epoxies for railway applications. Thus, PBF-a based composite materials can realize improvements in terms of lightweight design and simultaneously maintain high fire safety levels in, e.g., the railway industry without the need for further flame retardant additives.

## Figures and Tables

**Table 1:** Results of the spread of flame testing.

Material	CFE ^i^ (kW/m^2^)	Average Heat for Sustained Burning ^ii^ (MJ/m^2^)
**BFRP**	39 ± 3	4.3 ± 2.2
**GFRP**	34 ± 14	6.5 ± 2.7

^i^ incident heat flux at the surface of a specimen at the point along its horizontal centerline where the flame ceases to advance and may subsequently go out. ^ii^ averages of the values of heat for sustained burning, measured at a number of specified positions.

## Appendix A

**Table A1:** Physical properties of carbon, glass and basalt fiber reinforced polybenzoxazines.

Material-ID	No. of Plies	Thickness (mm)	FVC (vol%)	Density (g/cm^3^)
**CFRP**	20	4.08 ± 0.07	59.9 ± 0.3	1.5
**BFRP**	15	3.89 ± 0.05	56.7 ± 0.3	1.9
**GFRP**	14	4.21 ± 0.03	53.8 ± 0.6	1.9

**Table A2:** Mechanical properties of carbon, glass and basalt fiber reinforced PBF-a based polymers.

Material	Young´s Modulus (GPa)	Tensile Strength (MPa)	Elongation at Failure (%)	Flexural Modulus (GPa)	Flexural Strength (MPa)	Elongation at Failure (%)
**CFRP**	59 ± 2.3	788 ± 29	1.3 ± 0	47 ± 0.8	825 ± 32	1.9 ± 0.1
**BFRP**	32 ± 0.7	685 ± 28	2.6 ± 0.2	26 ± 0.8	666 ± 12	2.8 ± 0.1
**GFRP ^1^**	25 ± 0.7	411 ± 26 ^1^	2.1 ± 0.1	23 ± 0.3	687 ± 12	3.1 ± 0.1

^1^ Fracture at clamping.

**Table A3:** Parameters of the SN curves of BFRP and GFRP—For a Basquin’s fitting of log Sa = log So − 1/k*logN.

Material-ID	R	k	So	n	rsq
**BFRP**	−1.0	8.08	638.2	4	0.96
**BFRP**	0.1	8.96	277.7	7	0.94
**BFRP**	0.5	8.17	197.4	12	0.95
**GFRP**	−1.0	6.28	831.6	10	0.98
**GFRP**	0.1	6.96	374.1	8	0.95
**GFRP**	0.5	7.40	225.5	8	0.98

**Table A4:** Thermal decomposition of carbon, glass and basalt fiber reinforced PBF-a.

Material-ID	T_2wt%_ (°C)	T_10wt%_ (°C)	Char Yield (wt%)
**PBF-a**	283	374	43
**GFRP**	328	474	84
**BFRP**	324	471	84
**CFRP**	283	429	79

**Table A5:** Thermal-oxidative decomposition of carbon, glass and basalt fiber reinforced PBF-a.

Material-ID	T_2wt%_ (°C)	T_10wt%_ (°C)	Char Yield (wt%)
**PBF-a**	265	380	0
**GFRP**	359	496	71
**BFRP**	355	501	70
**CFRP**	262	467	27

**Table A6:** Cone calorimeter test results of neat PBF-a as well as carbon, glass and basalt fiber reinforced PBF-a.

Material	TTI (s)	PHRR (kW/m^2^)	THR (MJ/m^2^)	MARHE (kW/m^2^)
**PBF-a**	39 ± 1	353 ± 34	110 ± 1.1	200 ± 5.1
**CFRP**	78 ± 1	196 ± 9	34.9 ± 0.1	94.6 ± 1.7
**BFRP**	83 ± 47	132 ± 22	28.7 ± 2.7	49.7 ± 13.1
**GFRP**	59 ± 6	121 ± 20	33.4 ± 2.2	58.4 ± 2.3

**Table A7:** Smoke chamber test results of neat PBF-a as well as carbon, glass and basalt fiber reinforced PBF-a.

Material	Ignition Time (s)			D_S,max_ (/)	D_S_(4) (/)	VOF_4_ (/)
	Test 1	Test 2	Test 3			
**PBF-a**	550	550	/	702 ± 11	353 ± 13	598 ± 26
**CFRP**	192	164	/	430 ± 37	268 ± 20	287 ± 34
**BFRP**	170	No ignition	150	299 ± 52	224 ± 74	280 ± 117
**GFRP**	No ignition	No ignition	No ignition	342 ± 57	147 ± 51	150 ± 63

**Table A8:** Detected smoke gases in smoke chamber testing of neat PBF-a as well as carbon, glass and basalt fiber reinforced PBF-a.

Material	CIT_G_ (/)		Detected Gas Species	Amount of Gas Specie (ppm)	
	4 min	8 min		4 min	8 min
**CFRP**	0.08 ± 0.035	0.26 ± 0.010	CO_2_	3276 ± 1218	11842 ± 297
			CO	105 ± 23	333 ± 12
			HCN	7 ± 1.5	16 ± 1
			NO_X_	30 ± 13	96 ± 2.5
**BFRP**	0.07 ± 0.063	0.20 ± 0.129	CO_2_	2656 ± 2539	6614 ± 5752
			CO	132 ± 32	435 ± 119
			HCN	7 ± 2	21 ± 14
			NO_X_	41 ± 6	93 ± 5
**GFRP**	0.01 ± 0.007	0.06 ± 0.011	CO_2_	115 ± 55	364 ± 51
			CO	107 ± 54	495 ± 46
			HCN	7 ± 0	27 ± 6
**PBF-a**	0.02 ± 0.00	0.14 ± 0.01	CO_2_	187 ± 1.4	574 ± 8.5
			CO	338 ± 5.7	1194 ± 40
			HCN	14.3 ± 0.7	57 ± 1.8

## References

[1] Sapuan S.M., Sapuan S.M. (2017). Composite Materials. Composite Materials: Concurrent Engineering Approach.

[2] Feistauer E.E., Santos J.F., Amancio-Filho S.T. (2019). A review on direct assembly of through-the-thickness reinforced metal–polymer composite hybrid structures. Polym. Eng. Sci..

[3] Ghanta T.S., Aparna S.S., Verma N., Purnima D. (2020). Review on nano-and microfiller-based polyamide 6 hybrid composite: Effect on mechanical properties and morphology. Polym. Eng. Sci..

[4] Silvergleit M., Morris J.G., Larosa C.N. (1978). Flammability characteristics of fibre reinforced organic matrix composites. Polym. Eng. Sci..

[5] Kandola B.K., Horrocks A.R., Horrocks A.R., Price D. (2009). Chapter 5: Composites. Fire Retardant Materials.

[6] Hertzberg T. (2005). Dangers relating to fires in carbon-fibre based composite material. Fire Mater..

[7] Tang L., Zhang J., Tang Y., Zhou Y., Lin Y., Liu Z., Kong J., Liu T., Gu J. (2020). Fluorine/adamantane modified cyanate resins with wonderful interfacial bonding strength with PBO fibres. Compos. Part B Eng..

[8] Tang L., Dang J., He M., Li J., Kong J., Tang Y., Gu J. (2019). Preparation and properties of cyanate-based wave-transparent laminated composites reinforced by dopamine/POSS functionalized Kevlar cloth. Compos. Sci. Technol..

[9] Tang L., He M., Na X., Guan X., Zhang R., Zhang J., Gu J. (2019). Functionalized glass fibres cloth/spherical BN fillers/epoxy laminated composites with excellent thermal conductivities and electrical insulation properties. Compos. Commun..

[10] Liu Q., Shaw M.T., Parnas R.S., McDonnell A.-M. (2006). Investigation of basalt fibre composite mechanical properties for applications in transportation. Polym. Compos..

[11] Schürmann H. (2007). Werkstoffkunde der Faser-Kunststoff-Verbunde. Konstruieren mit Faser-Kunststoff-Verbunden.

[12] Gibson A.G., Mouritz A.P., Mouritz A.P., Gibson A.G. (2010). Thermal Decomposition of Composites in Fire. Fire Properties of Polymer Composite Materials.

[13] Gibson A.G., Mouritz A.P., Mouritz A.P., Gibson A.G. (2010). Health Hazards of Composites in Fire. Fire Properties of Polymer Composite Materials.

[14] Dhand V., Mittal G., Rhee K.Y., Park S.-J., Hui D. (2015). A short review on basalt fibre reinforced polymer composites. Compos. Part B Eng..

[15] Fiore V., Scalici T., Di Bella G., Valenza A. (2015). A review on basalt fibre and its composites. Compos. Part B Eng..

[16] Novitskii A.G. (2004). High-Temperature Heat-Insulating Materials Based on Fibers from Basalt-Type Rock Materials. Refract. Ind. Ceram..

[17] Hao L.C., Yu W.D. (2010). Evaluation of thermal protective performance of basalt fibre nonwoven fabrics. J. Therm. Anal. Calorim..

[18] Hao L., Yu W. (2011). Comparison of thermal protective performance of aluminized fabrics of basalt fibre and glass fibre. Fire Mater..

[19] Sawaryn C., Kreiling S., Schönfeld R., Landfester K., Taden A., Ishida H., Agag T. (2011). Benzoxazines for Industrial Applications Comparison with Other Resins, Formulation and Toughening Know-How, and Water-Based Dispersion Technology. Handbook of Benzoxazine Resins.

[20] Agag T., Geiger S., Ishida H. (2011). Thermal Properties Enhancement of Polybenzoxazines. Handbook of Benzoxazine Resins.

[21] Kourtides D.A., Gilwee W.J., Parker J.A. (1979). Thermal response of composite panels. Polym. Eng. Sci..

[22] Comer A., Ray D., Obande W., Clancy G., Rosca I., Stanley W. Out-of-Autoclave manufacturing of benzoxazine resin-systems by liquid-resin-infusion for ambient and high temperature aerospace application: Conference Paper. Proceedings of the 35th International Technical Conference & Forum; SAMPE.

[23] Jang J., Yang H. (2000). Toughness improvement of carbon-fibre/polybenzoxazine composites by rubber modification. Compos. Sci. Technol..

[24] Kiskan B., Ghosh N.N., Yagci Y. (2011). Polybenzoxazine-based composites as high-performance materials. Polym. Int..

[25] Shen S.B., Ishida H. (1996). Development and characterization of high-performance polybenzoxazine composites. Polym. Compos..

[26] Ishida H., Chaisuwan T. (2003). Mechanical property improvement of carbon fibre reinforced polybenzoxazine by rubber interlayer. Polym. Compos..

[27] Dillon F., Thomas K.M., Marsh H. (1993). The influence of matrix microstructure on the mechanical properties of CFRC composites. Carbon.

[28] Kimura H., Matsumoto A., Ohtsuka K. (2009). Glass fibre-reinforced composite based on benzoxazine resin. J. Appl. Polym. Sci..

[29] Ling H., Gu Y., Xie M. (2001). A New F-grade insulating glass cloth laminate based on benzoxazine. Insul. Mater..

[30] Xie M.L., Gu Y., Hu Z.R. (2000). Study on properties of polybenzoxazine/GF laminate. Insul. Mater. Commun..

[31] Zheng L., Zhang C., Cao Y., He Q., Gu Y. Study on blending resin of benzoxazine and properties of their laminates. Proceedings of the 15th National Composite Conference.

[32] Cal E., Maffezzoli A., Mele G., Martina F., Mazzetto S.E., Tarzia A., Stifani C. (2007). Synthesis of a novel cardanol-based benzoxazine monomer and environmentally sustainable production of polymers and bio-composites. Green Chem..

[33] Dansiri N., Yanumet N., Ellis J.W., Ishida H. (2002). Resin transfer molding of natural fibre reinforced polybenzoxazine composities. Polym. Compos..

[34] Tada Y., Nishioka Y., Takase H. (2004). Phosphazene compound, production method thereof and use of thereof.

[35] Liu F., Zhao X., Chen Y. (2008). Resin of phenolic modification benzoxazine and its properties of composite. Acta Mater. Comps. Sin..

[36] Yin C., Xiao J., Li J., Liu J., Zeng J., Jiang D. (2008). A study on quartz fibre reinforced benzoxazine resin composites. J. Natl. Univ. Defense Technol..

[37] Gu Y., Ran Q.-C., Ishida H., Agag T. (2011). Polybenzoxazine/Fibre Composites. Handbook of Benzoxazine Resins.

[38] Adams R.D. (1973). The effect of fibre diameter on the dynamic properties of glass-fibre-reinforced polyester resin. J. Phys. D Appl. Phys..

[39] Kun F., Carmona H.A., Andrade J.S., Herrmann H.J. (2008). Universality behind Basquin’s Law of Fatigue. Phys. Rev. Lett..

[40] Colombo C., Vergani L., Burman M. (2012). Static and fatigue characterisation of new basalt fibre reinforced composites. Compos. Struct..

[41] Zakaria K.A., Jimit R.H., Ramli S.N.R., Aziz A.A., Bapokutty O., Ali M.B. (2016). Study on fatigue life and fracture behaviour of fiberglass reinforced composites. J. Mech. Eng. Sci..

[42] Yin Y., Binner J.G.P., Cross T.E., Marshall S.J. (1994). The oxidation behaviour of carbon fibres. J. Mater. Sci..

[43] Kessler E., Gadow R., Straub J. (2016). Basalt, glass and carbon fibres and their fibre reinforced polymer composites under thermal and mechanical load. AIMS Mater. Sci..

[44] Mouritz A.P. (2006). Fire Safety of Advanced Composites for Aircraft.

[45] Hull T.R., Horrocks A.R., Price D. (2008). Challenges in fire testing: Reaction to fire tests and assessment of fire toxicity. Advances in Fire Retardant Materials.

[46] Gurit Composite Materials for Rail: Product Information. https://www.gurit.cn/-/media/Gurit/Datasheets/rail-materials-brochure.pdf.

[47] Horrocks A.R., Price D. (2008). Advances in Fire Retardant Materials.

